# Composite Ferroelectric Membranes Based on Vinylidene Fluoride-Tetrafluoroethylene Copolymer and Polyvinylpyrrolidone for Wound Healing

**DOI:** 10.3390/membranes11010021

**Published:** 2020-12-28

**Authors:** Tamara S. Tverdokhlebova, Ludmila S. Antipina, Valeriya L. Kudryavtseva, Ksenia S. Stankevich, Ilya M. Kolesnik, Evgenia A. Senokosova, Elena A. Velikanova, Larisa V. Antonova, Dmitry V. Vasilchenko, Georgiy T. Dambaev, Evgenii V. Plotnikov, Vyacheslav M. Bouznik, Evgeny N. Bolbasov

**Affiliations:** 1 Laboratory of Hybrid Plasma Systems, Tomsk Polytechnic University, Tomsk 634050, Russia; tst3@mail.tpu.ru (T.S.T.); vlk2@tpu.ru (V.L.K.); kss3@tpu.ru (K.S.S.); imk4@tpu.ru (I.M.K.); plotnikov.e@mail.ru (E.V.P.); 2 Department of Hospital Surgery with the Course of Cardiovascular Surgery, Siberian State Medical University, Tomsk 634050, Russia; Ant_sv@mail.ru (L.S.A.); ahhoo14@mail.ru (D.V.V.); dambaev@vtomske.ru (G.T.D.); 3 School of Engineering and Materials Science, Queen Mary University of London, London E1 4NS, UK; 4 Department of Chemistry & Biochemistry, Montana State University, Bozeman, MT 59717, USA; 5 Division of Experimental and Clinical Cardiology, Research Institute for Complex Issues of Cardiovascular Diseases, Kemerovo 650002, Russia; sergea@kemcardio.ru (E.A.S.); veliea@kemcardio.ru (E.A.V.); antolv@kemcardio.ru (L.V.A.); 6 Arctic Climate Materials Division, All Russian Scientific Research Institute of Aviation Materials, Moscow 105005, Russia; bouznik@ngs.ru; 7 Department of Inorganic Chemistry, Tomsk State University, Tomsk 634050, Russia

**Keywords:** ferroelectrics, composite, membranes, polyvinylpyrrolidone, wound healing

## Abstract

Wound healing is a complex process and an ongoing challenge for modern medicine. Herein, we present the results of study of structure and properties of ferroelectric composite polymer membranes for wound healing. Membranes were fabricated by electrospinning from a solution of vinylidene fluoride/tetrafluoroethylene copolymer (VDF–TeFE) and polyvinylpyrrolidone (PVP) in dimethylformamide (DMF). The effects of the PVP content on the viscosity and conductivity of the spinning solution, DMF concentration, chemical composition, crystal structure, and conformation of VDF–TeFE macromolecules in the fabricated materials were studied. It was found that as PVP amount increased, the viscosity and conductivity of the spinning solutions decreased, resulting in thinner fibers. Using FTIR and XRD methods, it was shown that if the PVP content was lower than 50 wt %, the VDF–TeFE copolymer adopted a flat zigzag conformation (TTT conformation) and crystalline phases with ferroelectric properties were formed. Gas chromatography results indicated that an increase in the PVP concentration led to a higher residual amount of DMF in the material, causing cytotoxic effects on 3T3L1 fibroblasts. In vivo studies demonstrated that compared to classical gauze dressings impregnated with a solution of an antibacterial agent, ferroelectric composite membranes with 15 wt % PVP provided better conditions for the healing of purulent wounds.

## 1. Introduction

Despite antibacterial treatment, infectious complications of wound continue to be a significant factor in contributing to patient morbidity and poor healing outcomes. Damaged skin cannot effectively perform protective, immune, respiratory, metabolic, thermoregulating, receptor, and other functions. It may provoke not only local complications, but also systemic pathological changes in the body [1]. 

Wound healing is a long-term multistage process, which may be inhibited by external environment [2]. Modern concepts of the pathophysiology of wound healing state several basic requirements for wound dressings including physical protection of wounds from external injury, inhibition of bacterial invasion, wound exudate absorption, maintaining physiological temperature and humidity, gas and liquid transport, mechanical flexibility, easy removal without adhesion to wounds, and biocompatibility [3]. These requirements are proven to prevent tissue dehydration and cell death, as well as improving intercellular interaction and angiogenesis and the wound healing process. Despite the variety of modern materials used for wound healing (foams, hydrogels, sprays, etc.) [4], electrospun polymer nonwowen membranes [5,6,7] meet all the necessary requirements [3] and are the most promising dressing material. 

Piezoelectric properties of collagen [8], the most common fibrillar protein that forms dermis, have triggered investigations directed towards devices and materials capable of accelerating tissue regeneration processes through electrical stimulation [9]. Ferroelectric and piezoelectric polymer materials and their application in reconstructive surgery have received closer attention [10]. Considerable interest in these materials and applications is due to the possibility of electrical stimulation of tissue regeneration processes via membrane mechanical deformations from cell, tissues, or organs [10]. This approach does not require external sources of electrical energy, batteries, or electrodes, excluding the possibility of accumulation of electrolysis products in tissues [11]. 

Due to the higher electronegativity of fluorine (F) compared to carbon (C) and hydrogen (H) in polyvinylidene fluoride (PVDF), which is the most electrically active piezoelectric polymer [12], and its copolymers with tetra-(TeFE) and trifluoroethylene (TrFE), a certain conformation of macromolecules results in a dipole moment in a polymer chain directed perpendicular to its axis. Currently, the application of PVDF elecrospun scaffolds as a wound healing material is being actively studied [13,14,15,16]. 

On the other hand, PVDF electrospun membranes suffer from rigidity and low flexibility due to the high hardness and elasticity modulus of PVDF [17] and can potentially cause damage to regenerated tissues interacting with the membrane. Heating PVDF membranes above 100 °C and subsequent cooling provokes the transition of the ferroelectric β crystalline phase to the paraelectric α phase [18], reducing the regenerative potential of the membranes as well as the range of possible sterilization methods. In addition, the high chemical resistance of PVDF [19] significantly limits the application of this membrane type for drug delivery to injury sites.

A promising strategy to improve the properties of piezoelectric polymer membranes for wound healing is developing a composite material based on the vinylidene fluoride–tetrafluoroethylene copolymer (VDF–TeFE) and polyvinylpyrrolidone (PVP) [20]. Compared to PVDF, the VDF–TeFE copolymer is characterized by a lower hardness and elastic modulus and a higher strength, relative elongation, crystallinity, pyroelectric coefficient, and electrostrictive constant, as well as the ability to form a ferroelectric β crystalline phase directly from the melt [21,22,23,24]. These properties along with high biocompatibility [25,26] make VDF–TeFE a polymer of choice for producing atraumatic membranes with high handling properties and electrical activity. The inclusion of nontoxic water-soluble PVP in the structure of VDF–TeFE membranes potentially allows encapsulation and efficient drug delivery to regeneration zones [27]. The development of ferroelectric composite membranes based on the VDF–TeFE copolymer is a growing area of research [20]. Thus, the influence of the PVP content on the VDF–TeFE/PVP composite is largely unclear. The properties of polymer solutions used for fabrication of composite membranes by electrospinning, membrane morphology and chemical composition, content of residual organic solvents, conformation of macromolecules and crystal structure of the VDF–TeFE copolymer in fabricated materials as well as the interaction of the membranes with cells and tissues in vitro and in vivo are needed to be investigated. This work aims to elucidate these questions and represents an important stage in comprehensive studies of the relationship between physicochemical and medico-biological properties of promising ferroelectric composite membranes based on the VDF–TeFE copolymer and PVP in order to evaluate the possibility of their use for wound healing.

## 2. Materials and Methods

### 2.1. Preparation of VDF–TeFE/PVP Membranes by Electrospinning

A VDF–TeFE copolymer according to GOST 25428-82 “Fluoroplast-42. Technical Specifications” (Halopolymer, Moscow, Russia), PVP (Kollidon® 90 F, BASF, Ludwigshafen am Rhei, Germany), and demitylformamide (DMF) (grade: pure according to GOST 20289-74 “Reagents. Dimethyl formamide. Specifications” (Ecros-1, Moscow, Russia) were used for solution preparation. Membranes were produced by electrospinning. Five types of spinning solutions were prepared with mass ratios of VDF–TeFE/PVP polymers of 100/0, 95/5, 85/15, 75/25, and 50/50. The total concentrations of polymers in solutions were 5 wt % for all samples. Polymers were dissolved in a sealed glass reactor at a temperature of 40 °C with constant stirring, until a homogeneous transparent solution was obtained. The resulting solution was cooled to the room temperature.

The viscosity of the spinning solutions was measured using the SV-10 viscometer (AND, Tokyo, Japan). The conductivity of the spinning solutions was measured using an InoLab Cond 7319 conductometer with a TetraCon 325 measuring cell (WTW, Weilheim, Germany). The viscosity and conductivity measurements of the spinning solutions were carried out at a temperature of 24 °C.

A commercially available NANON 01A electrospinning system (MECC Co., Fukudo, Japan), equipped with a cylindrical assembly collector with a diameter of 50 mm and a length of 200 mm, was used for the formation of nonwoven materials. The distance between a dope injector (22G needle) and an assembly manifold was 150 mm. The voltage at the injector was 25 kW. The flow rate of the dope solution was 1.8 mL/hour, and the assembly speed was 50 rpm. Membranes were fabricated at 20 °C and a relative humidity of 30%. 

### 2.2. Investigation of Physicochemical Properties of Membranes 

#### 2.2.1. Scanning Electron Microscopy (SEM)

The morphology of the samples was investigated by SEM using a JCM-6000 instrument (JEOL, Tokyo, Japan). Prior to the investigation, the samples were coated with a thin gold layer by a magnetron sputtering system SC7640 (Quorum Technologies Ltd., Lewes, UK). The fiber diameter was determined from SEM images captured in five fields of view using ImageJ 1.38 software (National Institutes of Health, Bethesda, MD, USA). The average diameter was determined from at least 120 fibers.

#### 2.2.2. X-ray Fluorescence Spectroscopy (XRF)

The study of the elemental composition of the samples was performed by XRF using the X-Ray fluorescence spectrometer XRF 1800 (Shimadzu, Kyoto, Japan) with an accelerating voltage of 40 kV, a current of 95 mA, a scanning speed of 8°/min, and a scanning step of 0.1°. Studies were conducted via a carbon (C), oxygen (O), fluorine (F), and nitrogen (N) channels. 

#### 2.2.3. Gas Chromatography (GC)

The study of the residual DMF content in the membranes was carried using the Kristall 5000 chromatograph with a flame-ionization detector (Khromatek, Novosibirsk, Russia) equipped with a ZB-5MS column (30 m × 0.25 mm × 0.25 μm). To determine the DMF content, 100 ± 5 mg of each sample were dissolved in 10.0 ± 0.1 mL of acetone. The following parameters were used: the volume of the injected sample, 1 μL; the evaporator temperature, 222 °C; detector temperature, 250 °C; thermostat temperature, 90 ° C; inlet pressure, 100 kPa; flow rate, 10 mL/min.

#### 2.2.4. Fourier-Transform Infrared Spectroscopy (FTIR)

The chemical composition and conformation of macromolecules VDF–TeFE in composite membranes were investigated using an attenuated total reflectance (ATR) FTIR system Tensor 27 (Bruker, Ettlingen, Germany) with ATR attachment PIKE MIRacle (Bruker, Ettlingen, Germany) on the crystal ZnSe. Studies were carried out in a spectral range of 650–2000 cm^−1^ with a resolution of 2 cm^−1^. The spectral data were processed in OPUS 3D (Bruker, Ettlingen, Germany) software.

#### 2.2.5. X-ray Diffraction Analysis (XRD)

The crystal structure of the VDF–TeFE copolymer in composite membranes was investigated using XRD 6000 (Shimadzu, Kyoto, Japan). The samples were exposed to a monochromatic Cu Kα (1.54056 Å) radiation. The accelerating voltage and the beam current were set to 40 kV and 30 mA, respectively. The scanning angle range, scanning step size, and signal collection time were 7°–30°, 0.0200°, and 2.5 s, respectively.

### 2.3. Investigation of Biomedical Properties of Membranes

#### 2.3.1. Adhesion, Viability, and Proliferative Activity of Cells

The study of the interaction of the obtained membranes with cells in vitro was carried out using mice embryo fibroblasts 3T3L1. Sterile polymers samples were placed into the wells of 24-well plates and filled by cell growth media at a ratio of 200 mg material weight per 1 mL of a medium. The samples were left for 5 days in a CO_2_ incubator at 37 °C for extraction. A medium without polymer disks was used as a control and kept in same conditions for 5 days. For all cells experiments, we used the same media DMEM (Gibco, Gaithersburg, MD, USA) supplemented with the glutamine supplement GlutaMAX (Gibco, Gaithersburg, MD, USA), 10% fetal bovine serum One Shot^®^, (Thermo Fisher Scientific, São Paulo, Brazil), and antibiotics (penicillin/streptomycin mixture) (Paneko, Moscow, Russia). After extraction, media were used for cell growth. The fibroblasts were previously seeded in 96-well plates, and after 24 h, the media were replaced by media with polymers extract. The cell were cultured for 24 and 120 h in an atmosphere containing 5% CO_2_ at a temperature of 37 °C and saturated humidity.

The absolute number of cells per 1 mm^2^ of the surface evaluated using a fluorescence microscopy system AxioVert.A1 (Carl Zeiss, Oberkochen, Germany). Cells were stained with vital fluorescent dyes Calcein AM 0.5 μg/mL (Abcam, Cambridge, Massachusetts, USA) for the green staining of cells cytoplasm and Hoechst 33342 1 μg/mL(Sigma-Aldrich, St. Louis, MI, USA) for the blue staining of nuclei of all adhered cells. The dyes were applied to the samples 15 min before microscopy. 

Image processing was performed using the ZEN pro software (Carl Zeiss, Oberkochen, Germany). Cells were counted using the ImageJ 1.38 software (National Institutes of Health, Bethesda, MD, USA) using 10 different fields of view. Studies were performed on 5 samples of each studied groups in triplicate using 10 randomly selected fields of view for each group. Cells cultured in media without material extraction were used as a control.

#### 2.3.2. In Vivo Contaminated Full-Thickness Wound Healing with Composite Membranes

The wound healing activity of membranes was studied in 20 adult Wistar rats (body weight: 180–200 g). Contaminated full-thickness wounds were formed. Rats were anesthetized, and a rectangular excision area (20 mm × 20 mm) was cut on each animal. The edges of the wounds and underlying muscles were crushed with Kocher’s forceps. After that, microbial suspension containing 10^6^ colony-forming unit (CFU) of *Staphylococcus aureus* was applied topically to the wound area. The surface of the wound was covered with a plastic wrap for 72 h to form an acute inflammation. Animals were divided into two groups with 10 animals in each group. For the animals of the first group, a gauze bandage soaked in an aqueous solution of chlorhexidine (Kemerovo pharmaceutical factory, Kemerovo, Russia) was applied to the wound surface. The dressings were changed on 3, 5, and 7 days of the experiment.

Macroscopic photographs EOS 250D digital camera (Canon, Tokyo, Japan) were used to evaluate the healing activity of membranes. For histological analyses, the tissue samples were harvested from the region of interest. The samples were fixed in formalin and processed for histopathological observation. The prepared 5 μm thick sections of tissues were stained with hematoxylin and eosin and studied using a transmission light microscopy system Axioscop40 (Carl Zeiss, Oberkochen, Germany). 

The study was carried out in accordance with the principles of humane treatment of laboratory animals described in [21]. Prior to investigation, all samples were sterilized in ethylene oxide atmosphere using a gas sterilizer AN4000 (Andersen Products Ltd, Clacton-on-Sea, UK).

### 2.4. Statistics

The data were analyzed with Origin 9.0 (OriginLab, Northampton, MA, USA) software using the one-way ANOVA with Tukey’s correction. The difference was considered significant at a significance level *p* of <0.05.

## 3. Results and Discussion

**Figure 1:**
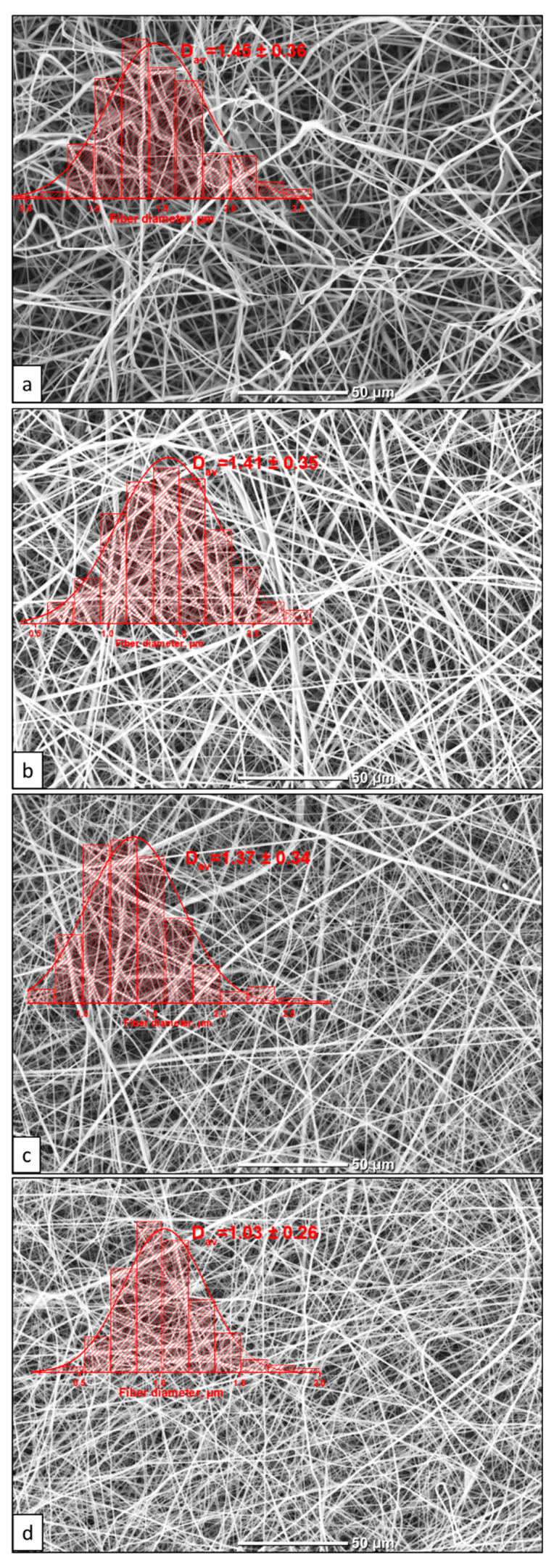
SEM images and fiber size distribution in the VDF–TeFE membranes with different PVP contents: (**a**) 0%; (**b**) 5%; (**c**) 15%; (**d**) 50%.

The morphology images of the formed VDF–TeFE membranes and composite membranes with different PVP contents are shown in Figure 1. Regardless of the PVP contents in the spinning solutions, all the membranes were formed by cylindrical fibers of regular shape, randomly intertwining with each other. However, an increase in the PVP content in the spinning solutions led to a decrease of the fiber diameter from 1.45 ± 0.36 to 1.03 ± 0.26 µm due to a decrease in the conductivity and dynamic viscosity of the solutions (Table 1). The observed decrease in the dynamic viscosity is attributed to the lower content of the polymer with a higher molecular weight in the mixture. Moreover, the solution conductivity drop from 2.84 ± 0.02 to 1.50 ± 0.04 μS/cm, accompanied by a decrease of the spinning solution viscosity, was probably induced by intermolecular interaction, leading to the formation of complexes between the polymers and the solvent. 

**Figure 2:**
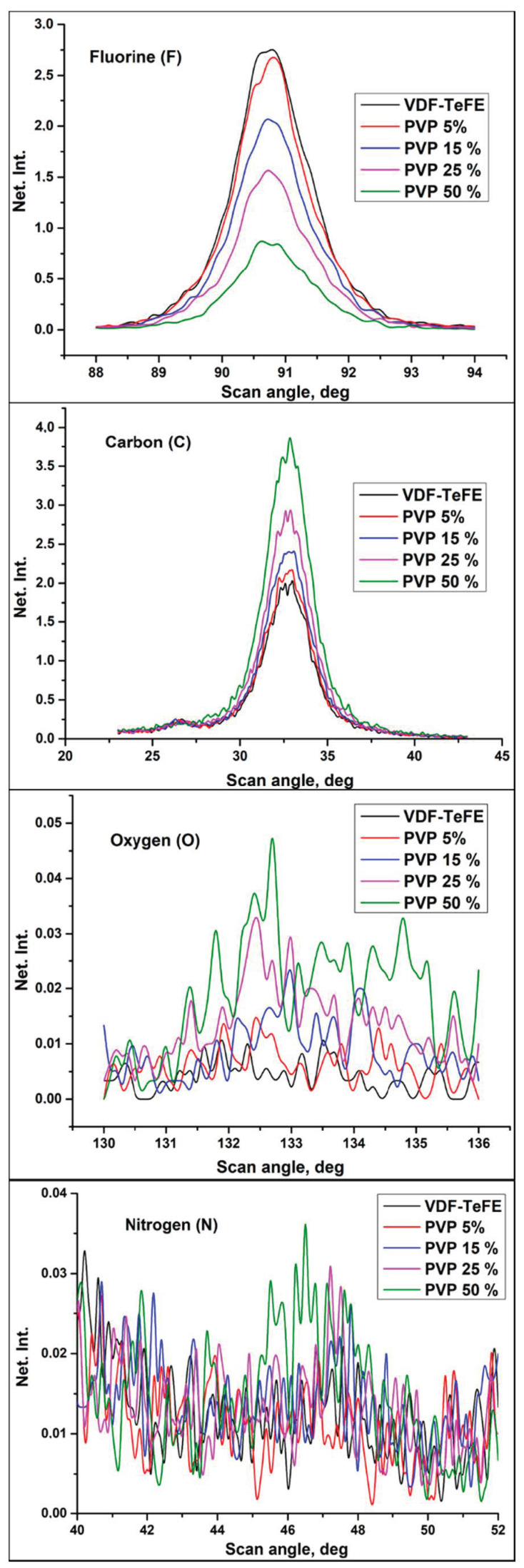
Fluorescent spectra in the VDF–TeFE membranes with different PVP contents.

Figure 2 shows the typical fluorescence spectra of the investigated samples for the following elements: carbon (C), oxygen (O), fluorine (F), and nitrogen (N). The chemical composition of the VDF–TeFE membranes without PVP was mainly represented by carbon (C) and fluorine (F), which are major elements in VDF–TeFE backbone. Low amount of oxygen (O) may be attributed to the presence of residual DMF used as a solvent for the preparation of the spinning solutions (Table 2).

An increase of the PVP concentration in the spinning solutions led to an increase in the content of oxygen, nitrogen, and carbon in the formed membranes (Table 2). These elements were presented in the composition of both PVP and DMF. The fluorine content in the formed membranes decreased, which is evidenced by the formation of a polymer composite. At the same time, an increase of the PVP amount led to a higher DMF content in the samples (Table 2). Probably, the observed increase in the DMF content in the formed composite samples, as well as a decrease in the conductivity of the spinning solutions, is evidence of the formation of the complex between PVP and DMF [28].

The ferroelectric properties of PVDF and its copolymers were determined by the conformation of macromolecules and by its crystalline structure. There are three main polymorphs (α, β, and γ). The α phase is characterized by a monoclinic lattice in which a TGTG^−^ chain conformation has opposite dipole moments, so in general it is nonpolar. The γ phase contains a weakly polar cell with a T3GT3G^−^ chain conformation. The β phase is the most electroactive and is characterized by orthorhombic lattice with polar cells in which the chain has a planar zigzag (TTT) conformation [18]. The presence of polymorphic conformations and crystal structures typical of the paraelectric and ferroelectric phases allows determining their presence, using techniques such as FITR and XRD [29].

**Figure 3:**
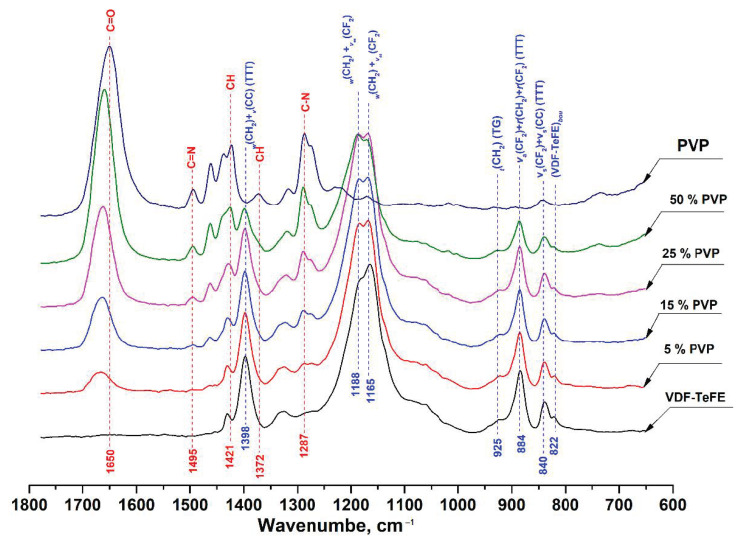
FTIR spectra of composite VDF–TeFE/PVP membranes with different PVP contents.

The FTIR spectra of the studied VDF–TeFE membranes with different PVP contents are shown in Figure 3.

Starting with the FTIR spectrum of pure PVP, the absorption band located around 1650 cm^−1^ can be ascribed to the stretching vibration of C=O in the pyrrolidone group. The bands at 1421 and 1372 cm^−1^ corresponded to the CH deformation modes from the CH_2_ group [30]. The peaks located at 1287 cm^−1^ are related to the C–N bending vibration from the pyrrolidone structure. The small absorption band at about 1495 cm^−1^ referred to the characteristic vibration of C=N (pyridine ring) [31].

In the spectrum of a membrane made of the VDF–TeFE copolymer, two intense absorption bands were observed in the regions of 1165 and 1188 cm^−1^, which were superpositions of twisting and stretching vibrations in the CH_2_ and CF_2_ groups, respectively. The intense absorption band located at 1398 cm^−1^ corresponded to a superposition of wagging and antisymmetric stretching vibrations in the CH_2_ and C–C groups. The band in the 884 cm^−1^ region was a superposition of antisymmetric stretching and rocking vibrations in the CF_2_ group and rocking vibrations in the CH_2_ group. The insignificant band in the region of 840 cm^−1^ was a superposition of symmetric stretching vibrations in the CF_2_ and C–C groups [32,33]. 

The presence of the intense bands in the regions of 840, 884, and 1398 cm^−1^ corresponding to the transconformations with a slight band in the region of 925 cm^−1^ gauche conformation indicated that macromolecules of the VDF–TeFE copolymer in the formed membranes had predominantly a planar zigzag conformation with a strong dipole moment directed perpendicular to the axis of the polymer chain [34]. 

With an increase of the PVP content in the membranes, increases in the intensity of the bands located at 1650, 1495, 1421, 1372, and 1287 cm^−1^ were observed, whereas the intensities of the bands located at 1165, 1188, 1398, 884, and 840 cm^−1^ corresponding to the VDF–TeFE copolymer decreased. An increase in the PVP content in the formed membranes retained the conformation of a planar zigzag in the macromolecules of the VDF–TeFE copolymer, as evidenced by the presence of the bands in the region of 840, 884, and 1398 cm^−1^ as well as the absence of shifts of these bands, regardless of the PVP contents in the formed membranes. In addition, in the composite membranes, a shift of the band from the region of 1650 cm^−1^ (C=O in the pyrrolidone group) to the region of 1662 cm^−1^ was observed. Such a shift may indicate dipole–dipole interactions between PVP and VDF–TeFE [35].

**Figure 4:**
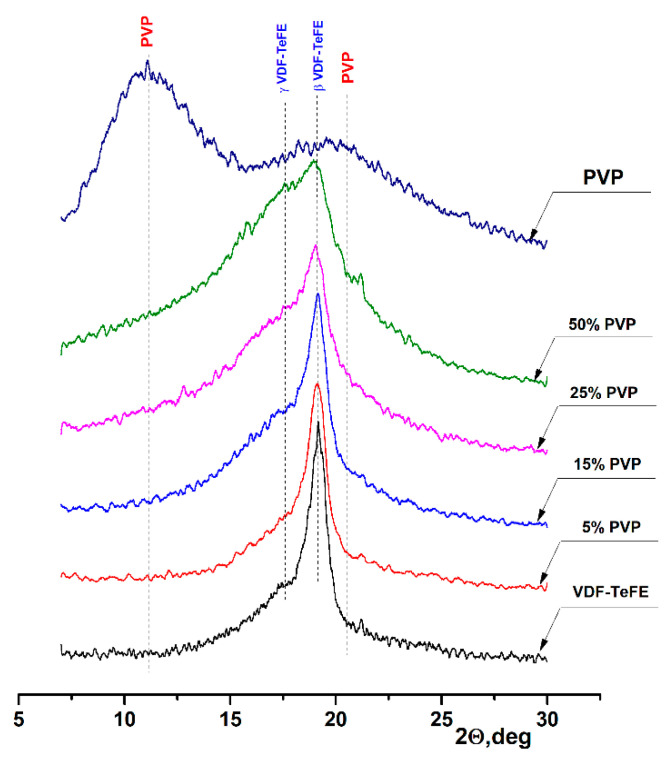
XRD patterns of the composite VDF–TeFE/PVP membranes with different PVP contents.

The XRD patterns of the formed membranes are shown in Figure 4. The XRD pattern of the pure PVP shows two wide characteristic halo peaks located at 10.9° and 20.5°, corresponding to the amorphous PVP [36]. An intensive peak at 19.2° on the XRD pattern of the membranes formed from the VDF–TeFE copolymer corresponded to the most electrically active ferroelectric β phase of the VDF–TeFE copolymer formed by macromolecules in TTT conformation [37,38]. At the same time, a halo associated with the formation of an electrically active γ phase formed by macromolecules in the T3GT3G- conformation could be observed at 17.8° [39,40]. The formation of electrically active crystalline phases is associated with the high intensity of the electric field between the injector and the collector, high tensile forces from the electric field in the process of the membranes formation [41], and the alignment effect of TeFE segments on the PVDF macromolecule [42]. With an increase of the PVP content, the half-width of the β phase peak increased from 0.784° for the pure VDF–TeFE membranes to 1.952° for the VDF–TeFE membranes containing 50% PVP. The intensity of the halo reflection at 17.8° also increased, indicating difficulties of the electrically active β phase crystallization process in the VDF–TeFE copolymer. It can be attributed to the penetration of PVP molecules into the VDF–TeFE structure and the formation of significant transition layers formed as a result of tensile forces from the electric field. 

As the PVP concentration in the VDF–TeFE-based composite membranes increased, the lower content of the electrically active β phase and its substitution by the γ phase was observed. This deteriorated the piezoelectric properties of the composite, since the values of piezoelectric and pyroelectric coefficients for the β phase are 1.5 ÷ 2 times higher than for the γ phase [43]. In turn, less pronounced piezoelectric properties reduce the ability of membranes to stimulate tissue regeneration processes. Our studies demonstrated that, in the composite membranes with a PVP content of 25%, the crystallization of the VDF–TeFE copolymer was significantly hindered. Thus, the optimal content of PVP, ensuring the efficient formation of the most electrically active β phase, was in a range of 15–25 mass%.

**Figure 5:**
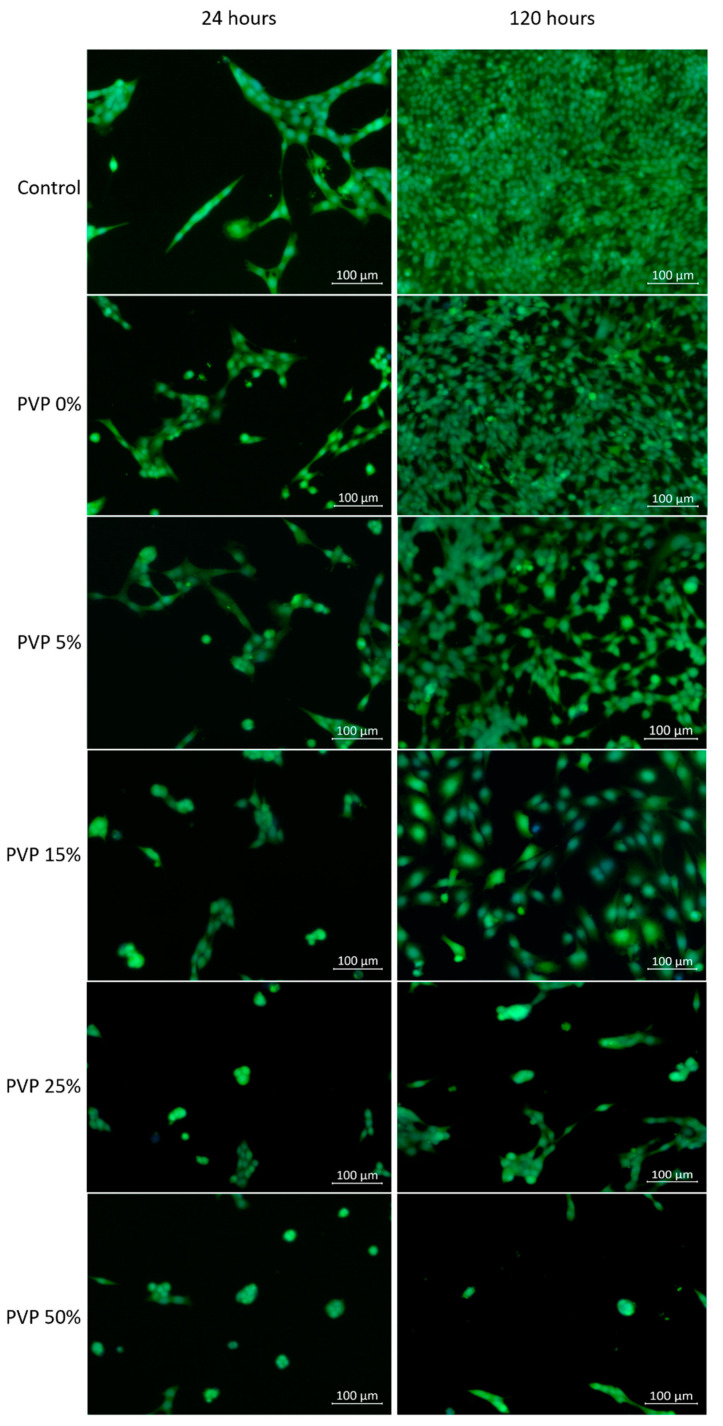
Images of cells on the surface of cell culture plates depending on the PVP content and culture period.

The images of cells on the surface of the cell culture plastic in the untreated media and media extract of the VDF–TeFE membranes with different PVP contents are shown in Figure 5. The number of fibroblasts adhered to the surface of the cell culture plastic decreased dramatically in dependence of the PVP content (Table 3). The highest number of cells adhered to membrane surface was observed for the VDF–TeFE membranes with a PVP content of 0 and 5 wt %. These samples were characterized by high viability and proliferation of adhered cells, which were similar to the control group. A significant decrease in the number of adherent cells for membranes started with a PVP content of 15 wt % and higher. PVP contents of 25 and 50 wt% or more led to a decrease of proliferation of fibroblasts (Table 3). These differences were not so significant at the 24 h point, but it became obvious after 120 h incubation.

The decrease in adhesion and proliferation of fibroblasts with an increase in PVP content may be due to the following reasons. PVP is a water-soluble, hydrophilic polymer. Thus, PVP can diffuse into the culture medium [44]. The amount of PVP released into the culture medium over a certain period of time is determined by the content of PVP in the sample. It is known that PVP is protonated in aqueous solutions and can form complexes with anions, including various biologically active molecules [45]. Thus, an increase of PVP content may lead to an increase in the content of complexes with biologically active anions in the culture medium, changing its chemical composition and reducing the availability of anions for cells. On the other hand, diffused PVP in the culture medium can lead to its accumulation in cells causing “lysosomal storage disease”, which inhibits the processes of vital activity of cells causing their death [46]. The third possible reason for a decrease in cell viability on the surface of membranes with a high content of PVP is the process of its leaching from fibers, leading to membrane deformation which prevents cell attachment to the membrane surface [46]. The fourth reason could be the formation of toxic complexes between PVP and DMF during the spinning solution preparation, followed by its migration into the culture medium, which is indirectly evidenced by an increase in the concentration of DMF in the formed membranes with an increase in the concentration of PVP (Table 2). 

The conducted studies showed that an increase in the content of PVP in the formed membranes above 25–50% did not significantly change the structure of the formed membranes, but it hindered the crystallization of the VDF–TeFE copolymer in the most electrically active β phase and negatively affected the viability of 3T3L1 fibroblasts. The composite membranes containing 15 wt % PVP had an optimal morphology, chemical, crystal structure and were sufficiently capable of maintaining the necessary conditions for adhesion and proliferation of fibroblasts, which makes it possible to be used for pilot studies to explore the possibility of their use to restore the skin in the case of contaminated full-thickness wounds. 

**Figure 6:**
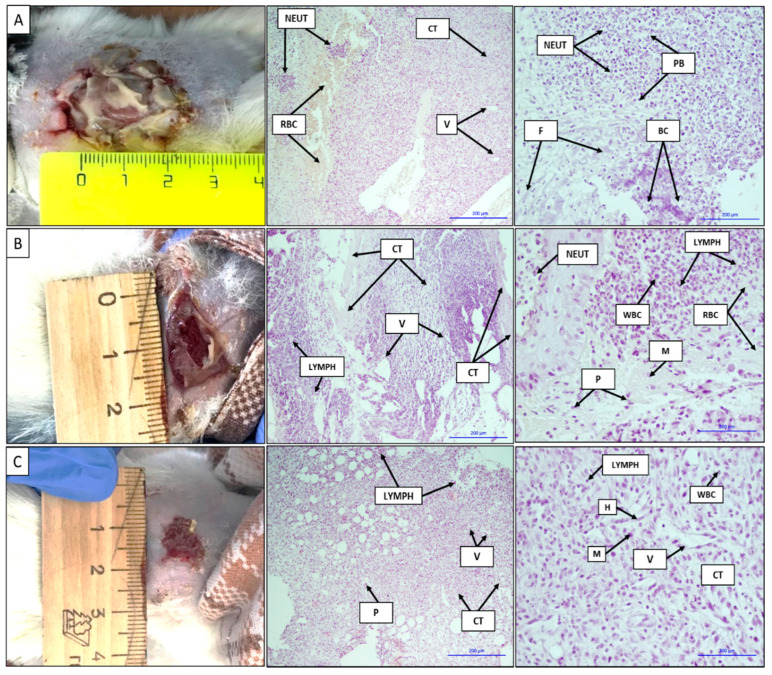
Images and histological sections of the contaminated full-thickness wound (**A**), the wound treated with a gauze dressing soaked in a chlorhexidine solution (**B**), and the wound treated with the VDF–TeFE membrane containing 15 wt % PVP on the 7th day (**C**). V: vessels, H: histiocytes, NEUT: neutrophils, WBC: leukocytes, RBC: erythrocytes, CT: connective tissue, LYMPH: lymphocytes, M: macrophages, P: plasma cells, F: fibrin, purulent bodies (PB): purulent bodies, bacterial colonies (BC): colonies of bacteria. The bars correspond to 200 μm.

The images and histological sections of the contaminated full-thickness wound, wound treated with a gauze dressing soaked in a chlorhexidine solution, and wound treated with the VDF–TeFE membrane containing 15 wt % PVP after 7 days are shown in Figure 6. The formed contaminated wound is a focus of acute purulent-necrotic inflammation, as evidenced by the gray-green color accompanied by a characteristic unpleasant odor on the surface of the wound. The shape of the wound was circular with a diameter of ~2.5 cm (Figure 6A). Purulent inflammation with purulent bodies and a large number of neutrophils hemorrhage foci were observed on the histological sections. Basophilically stained structureless colonies of microorganisms were subtotally determined. Neutrophil infiltration into adipose tissue, erythrocytes, thin fibrin fibers, connective tissue with moderate lymphoid infiltration with the presence of leukocytes and neoangiogenesis, and vasculitis and erythrocytes in the vascular lumen were observed (Figure 6A).

The wound size treated with a gauze bandage soaked in a chlorhexidine solution decreased to 1.5 cm on the 7th day of the experiment. The bottom of the wound was covered with an abundant layer of fibrin, easily separated from the wound upon contact. An active formation of granulation tissue was observed under the fibrin layer (Figure 6B). On the histological sections, mature and immature connective tissues with a pronounced lymphoid infiltration and the presence of vascular fissures were observed. On the periphery of the histological sections, an inflammation in the adipose tissue, represented by a large number of neutrophils with masses and filaments of fibrin, as well as bacterial colonies, was observed. There was a pronounced neutrophilic infiltration with the presence of lymphoid cells and macrophages. On the left side of the image, a layer of stratified squamous epithelium with edema and the presence of neutrophils on its surface can be seen (Figure 6B).

In case of the VDF–TeFE membrane containing 15% PVP wound treatment, the diameter of the wound decreased up to ~1 cm on the 7th day of the experiment. A slight granulation tissue appeared on the surface of the wound. Active epithelialization and tissue granulation were observed on the periphery of the wound. Histological sections were represented by fibrous adipose tissues, pronounced neoangiogenesis, and moderate lymphoid infiltration. Among the cellular infiltrate, single plasmocytes were determined. In the fibrous tissue, a heterogeneous cellular infiltrate was clearly defined, represented mainly by lymphohistiocytes and to a lesser extent by plasma cells, macrophages, and neutrophils. Fibrous adipose tissues with a moderate lymphoid infiltration and the presence of plasma cells and single neutrophils, as well as vessels with an endothelial lining, were observed (Figure 6C).

The VDF–TeFE membrane containing 15% PVP showed a better effect on skin healing in the case of the contaminated full-thickness wound compared to standard gauze dressings impregnated with chlorhexidine. It might be due to following reasons. First, membranes made by electrospinning have interconnected porosity formed by micron-sized fibers, resulting in a significant free surface [47] and the ability of PVP to bind toxins and water [45]. These properties of the obtained membranes make it possible to absorb a significant amount of exudate released by pathogenic microflora, as well as maintaining the required moisture level on the wound surface. The absorbed exudate saturated with pathogens is removed along with the membrane, thus reducing the concentration of pathogens with each subsequent dressing. In addition, PVP released from fibers into the exudate can accumulate in pathogenic cells, causing “lysosomal storage disease” to provoke their death [48]. Thus, the presence of PVP in fibers is a factor that reduces the concentration of pathogens in the regeneration zone. 

Second, it is known that PVDF-based piezoelectric polymer membranes are capable of negatively affecting the *Staphylococcus* culture when under a dynamic load of the membrane, even in the absence of antibacterial agents [49]. Since in membranes containing 15 wt % of PVP the predominant conformation of VDF–TeFE macromolecules was the electrically active trans conformation (Figure 2) and crystallites with characteristic ferroelectric properties predominate in the crystal structure (Figure 3), it can be assumed that the decrease in the concentration of pathogens in the wound and improved tissue regeneration is due to the piezoelectric properties of the formed membranes. 

Third, it is known that, under the influence of external mechanical stimuli, piezoelectric membranes can enhance migration, adhesion, and cytokine secretion in NIH3T3 fibroblasts in vitro [50]. The capacity of piezoelectric membranes to generate electrical impulses in response to the mechanical action of the tissues surrounding the implant allows promoting the wound healing, regardless of the implantation zone [51]. Thus, the piezoelectric properties of the formed membranes make it possible to enhance the processes of tissue regeneration. 

## 4. Conclusions

Composite VDF–TeFE/PVP membranes with different PVP contents have been successfully fabricated by electrospinning. It has been shown that, regardless of the PVP concentration, the membranes were formed by randomly intertwining fibers, providing interconnected porosity. An increase in the PVP content in spinning solutions led to a decrease in the fiber diameter due to the lower conductivity and dynamic viscosity of the solutions. Higher PVP concentration also resulted in an increase in the content of oxygen (O) and nitrogen (N) in the formed membranes while hindering the crystallization process of the VDF–TeFE copolymer in the ferroelectric β phase.

The cytotoxicity and proliferative activity of human fibroblasts cultured on the membranes surface decreased with an increase in PVP amount. It has been shown that the optimum PVP content in the spinning solution was 15 wt %. Studies of the healing process of contaminated full-thickness wounds showed that, compared to standard gauze dressings soaked in an aqueous solution of chlorhexidine, composite VDF–TeFE/PVP membranes demonstrated a better healing effect.

## Figures and Tables

**Table 1:** Dynamic viscosity values, conductivity values of spinning solutions, and mean fiber diameters in the formed membranes.

Polyvinylpyrrolidone (PVP; %)	Dynamic Viscosity, (mPa·s)	Conductivity (µS/cm)	Mean Fiber Diameter (µm)
0	845 ± 11	2.84 ± 0.02	1.45 ± 0.36
5	725 ± 14	2.66 ± 0.02	1.41 ± 0.35
15	622 ± 7	2.42 ± 0.03	1.37 ± 0.34
25	492 ± 16	2.06 ± 0.02	1.30 ± 0.32
50	227 ± 9	1.50 ± 0.04	1.03 ± 0.26

**Table 2:** Elemental compositions of vinylidene fluoride/tetrafluoroethylene (VDF–TeFE) membranes with different PVP contents.

PVP Content (%)	Elemental Composition (at. %)				Residual Dimethylformamide (DMF; ppm)
	C	O	F	N	
0	36.5 ± 0.3	1.4 ± 0.2	62.1 ± 0.2	-	762 ± 48
5	38.0 ± 0.3	1.8 ± 0.2	60.2 ± 0.4	-	1195 ± 98
15	42.3 ± 0.2	3.1 ± 0.4	54.7 ± 0.2	-	1521 ± 75
25	42.1 ± 1.3	4.3 ± 0.6	45.5 ± 0.4	8.1 ± 1.1	1912 ± 120
50	50.7 ± 0.4	6.9 ± 0.7	30.1 ± 0.4	11.4 ± 1.1	2423 ± 136

**Table 3:** Cell growth and proliferation after 24 and 120 h of incubation.

Specimens Names	Number of Fibroblasts (pcs/mm^2^), Me (Q1;Q3)	
	24 h	120 h
Control	240 (175;291)	1214 (980;1312)
PVP content (%)		
0	220 (182; 232) *	1112 (967;1290)
5	146 (83; 187) *	790 (693;843)
15	92 (69; 102) *,**	376 (198;452)
25	35 (9.0;37.0) *,**	158 (40;172)
50	27 (5.5;33.0) *,**	67 (34;76)

* *p* < 0.05 compared to control, ** *p* < 0.05 compared to the VDF–TeFE membranes with a PVP content of 0%.
